# Reduction of hypokalaemia with finerenone: a drug or class-specific effect?

**DOI:** 10.1093/ehjcvp/pvae089

**Published:** 2025-01-08

**Authors:** Giuseppe M C Rosano, Koh Ono, Egidio Imbalzano, Koji Hasegawa

**Affiliations:** Department of Human Sciences and Promotion of Quality of Life, Chair of Pharmacology, San Raffaele University of Rome, via di val cannuta, Rome 00163, Italy; Cardiology, San Raffaele Cassino Hospital, Via Gaetano di Biasio, 1, Cassino FR 03043, Italy; Department of Cardiovascular Medicine, Kyoto University Graduate School of Medicine, Kyoto 606-8507, Japan; Department of Internal Medicine, University of Messina, Via Consolare Valeria Cap, Messina 98125, Italy; National Hospital Organization Kyoto Medical Centre, 1-1 Mukaihaya-cho, Fukakusa, Fushimi-ku, Kyoto 612-8555, Japan

**This editorial refers to ‘Hypokalaemia in patients with type 2 diabetes and chronic kidney disease: the effect of finerenone—a FIDELITY analysis’, by B. Pitt *et al.*  https://doi.org/10.1093/ehjcvp/pvae074.**

The interplay between chronic kidney disease (CKD), type 2 diabetes (T2D), and cardiovascular health is well known. In recent years, the attention has been drawn to the occurrence of hyperkalaemia in patients with cardio-renal-metabolic diseases (CRMD), mostly because of the advent of the new potassium binders, but the recent analysis by Pitt *et al.* of the FIDELITY pooled analysis of the FIDELIO-DKD and FIGARO-DKD trials, focuses on the critical aspect of hypokalaemia and cardiovascular outcomes.^1^

Hypokalaemia, defined as serum potassium levels below 3.5 mmol/L, has long been recognized as a potential herald of cardiovascular complications, and it is associated with cardiovascular risk.^2^ The FIDELITY analysis brings into focus a concerning prevalence of this condition among CKD patients with T2D. The analysis surprisingly demonstrated that 41.1% of patients with diabetes and CKD experienced hypokalaemia with serum potassium <4.0 mmol/L, while 7.5% experienced more severe hypokalaemia with levels <3.5 mmol/L. These figures underscore the prevalence of potassium imbalance in this vulnerable population.

More alarmingly, the analysis demonstrated a clear association between lower baseline serum potassium levels and increased risk of adverse cardiovascular outcomes. Patients with baseline serum potassium <4.0 mmol/L faced a 16% higher risk of the cardiovascular composite outcome, and a 20% higher risk of the arrhythmia composite outcome compared to those with levels between 4.0 and 4.5 mmol/L. These findings reinforce the critical importance of maintaining optimal potassium balance in CKD patients, particularly those with concomitant T2D.

The FIDELITY analysis provides compelling evidence for the potential benefits of the selective mineralocorticoid receptor antagonist (MRA) finerenone on hypokalaemia in patients with CRMD. The study demonstrated that finerenone treatment significantly reduced the incidence of hypokalaemia compared to placebo, with a 37% reduction in cases of serum potassium <4.0 mmol/L and a 54% reduction in cases of serum potassium <3.5 mmol/L. Finerenone was also associated with a reduced hazard of both cardiovascular and arrhythmia events across all baseline serum potassium subgroups. This suggests that the cardioprotective effects of finerenone extend beyond its impact on potassium homeostasis, potentially offering a multifaceted approach to managing cardiovascular risk in CKD patients with T2D.

While the findings regarding finerenone are promising, it is crucial to place these results within the broader landscape of MRAs therapies. Other MRAs such as spironolactone and eplerenone have long been the cornerstones in the management of heart failure and resistant hypertension. However, these drugs are also known to affect potassium homeostasis, and they have been long used as potassium sparing diuretics. While pharmacodynamic differences suggest potentially unique effects of finerenone, direct head-to-head comparisons with other MRAs are still lacking. Such studies would be valuable to definitively establish the relative efficacy and safety profiles of these drugs.

The apparent paradox between the hypokalaemia-reducing effects of finerenone and the tendency of MRAs to increase potassium levels highlights the complex relationship between these drugs and electrolyte balance. It underscores the need for careful monitoring and individualized treatment approaches when using any MRA therapy in patients with CKD and T2D.^3^

The findings of the FIDELITY analysis have several important implications for clinical practice. First, the high prevalence of hypokalaemia in CKD patients with T2D emphasizes the need for regular and careful monitoring of serum potassium levels in this population and the effect of finerenone on hypokalaemia highlights the central role of MRAs in the management of cardiovascular risk in this patient population.

However, there are several questions that deserve further investigation. We do not know yet what are the precise mechanisms by which finerenone exerts its cardioprotective effects, and how do these differ from the non-steroidal MRAs and whether there are different long-term cardiovascular and renal outcomes of finerenone compared to steroidal MRAs.

The FIDELITY analysis is an important reminder of the often-overlooked threat of hypokalaemia in CKD patients with T2D and in patients with heart failure. It highlights the effect, albeit not unique, of finerenone in this space while also underscoring the complex interplay between MRAs, electrolyte balance, and cardiovascular outcomes. Managing cardiovascular risk in CKD patients with T2D requires a multifaceted approach in which the management of optimal potassium balance is important but is just one piece of a larger effect that includes a significant effect on cardiovascular events. The findings of this study suggest the need to be vigilant in monitoring and managing electrolyte imbalances in CKD patients with T2D and in patients at increased risk of heart failure. They also underscore the effect of finerenone in reducing hypokalaemia. This effect is not unique to finerenone and is also shared by steroidal MRAs. However, the effect of finerenone in these patients is not limited to its effect on hypokalaemia but extends to a more comprehensive cardiovascular protection.
